# Comparison of suturing models: the effect on perception of basic surgical skills

**DOI:** 10.1186/s12909-021-02692-x

**Published:** 2021-05-01

**Authors:** Alejandro Rafael Gonzalez-Navarro, Alejandro Quiroga-Garza, Adriana Sharai Acosta-Luna, Yolanda Salinas-Alvarez, Javier Humberto Martinez-Garza, Oscar de la Garza-Castro, Jorge Gutierrez-de la O, David de la Fuente-Villarreal, Rodrigo Enrique Elizondo-Omaña, Santos Guzman-Lopez

**Affiliations:** 1 Departamento de Anatomia Humana. Francisco I. Madero and Jose E. Gonzalez sin número, Colonia Mitras Centro Monterrey, Universidad Autónoma de Nuevo León, Facultad de Medicina, 64460 Monterrey, Nuevo León Mexico; 2 Instituto Mexicano del Seguro Social, Delegación de Nuevo Leon, General Surgery, Monterrey, Nuevo Leon Mexico

**Keywords:** Surgical skills, Suturing model, Suturing, Medical student training, Surgical training, Education, High-Fidelity models, Low-Fidelity models, Medical student

## Abstract

**Background:**

Acquisition of Basic Surgical Skills (BSS) are essential for medical students. The objective was to determine it’s fidelity impact.

**Methods:**

Using four suturing models (SM) (pigskin, sponge, commercial pad, and orange), SM-quality and student-SM interaction were evaluated. After a 1-h class, participants were divided into groups and randomly assigned exercises in SM in 15-min intervals. The experiment included completing three individual simple stitches and a 3-stitch continuous suture in each SM.

**Results:**

Eighty-two medical students participated. Suturing quality was better in pigskin and sponge, which were also the preferred models (*p* < 0.001). Significant differences in quality between the insertion and exit point, and firmness of knots (*p* < 0.05) in both simple and continuous sutures, as well as between length and distance in continuous ones (*p* < 0.001) were identified.

**Conclusions:**

Acquisition and quality of BSS are influenced by the intrinsic characteristics of SM. An adequate degree of resistance, consistency, and elasticity are necessary.

**Supplementary Information:**

The online version contains supplementary material available at 10.1186/s12909-021-02692-x.

## Background

Basic surgical skills (BSS) are generally taught during the early stages of medical school. These are fundamental competencies for any physician, that include various types of techniques such as gowning and gloving, drain insertions, infiltrating anesthetics, knot tying, incisions/excisions, and suturing wounds. Suturing is considered one of the cornerstones of medical student training and an essential competence of any surgical-oriented specialty [1, 2].

Ideally, students should dominate basic skills before working on patients, although this is not always the case in all countries due to a lack of formal training courses or resources. This creates an ethical and medico-legal conflict, which schools must emphasize. BSS is an ideal field for the application of simulation-based training. Knowledge and practice provide students with the confidence and skills needed to avoid iatrogenic errors and reduce anxiety [3, 4].

Suturing is one of the most commonly taught skills. Different teaching methods and materials have been described for suturing training. The use of vegetables [5, 6], animal parts [7, 8], synthetic materials [9], and commercial platforms have been applied as suturing models (SM) for this purpose. These can be used in surgical training laboratories with controlled environments that can resemble the in-hospital scenario with different degrees of accuracy [10]. Human anatomy laboratories are an ideal setting for BSS with the use of cadavers among a variety of other bench model simulations.

SM vary widely in regards to their level of fidelity or realism to living human tissue [11]. It’s important to consider the characteristics of each model, such as density, elasticity, rigidity, volume, viscosity, shear strength, wear resistance, among others when choosing one. Cost/benefit should also be evaluated, as well as the perception by the user. The purpose of this study is to determine the characteristics of different SM and their impact on BSS training (specifically to suturing skills) and student perception.

## Materials and methods

### Study design

A prospective, longitudinal, pre-experimental, and randomized study was designed. Announcements were made in the medical school to voluntarily recruit students from any year, regardless of suturing experience or knowledge, for study participation. Students currently enrolled in the medical degree program (a 12-semester program) were included. Those under 18 years of age, enrolled in another health science degree, or with upper extremity motor/coordination limitations were excluded. Those who did not sign or withdrew informed consent were eliminated.

### Materials

**Fig. 1:**
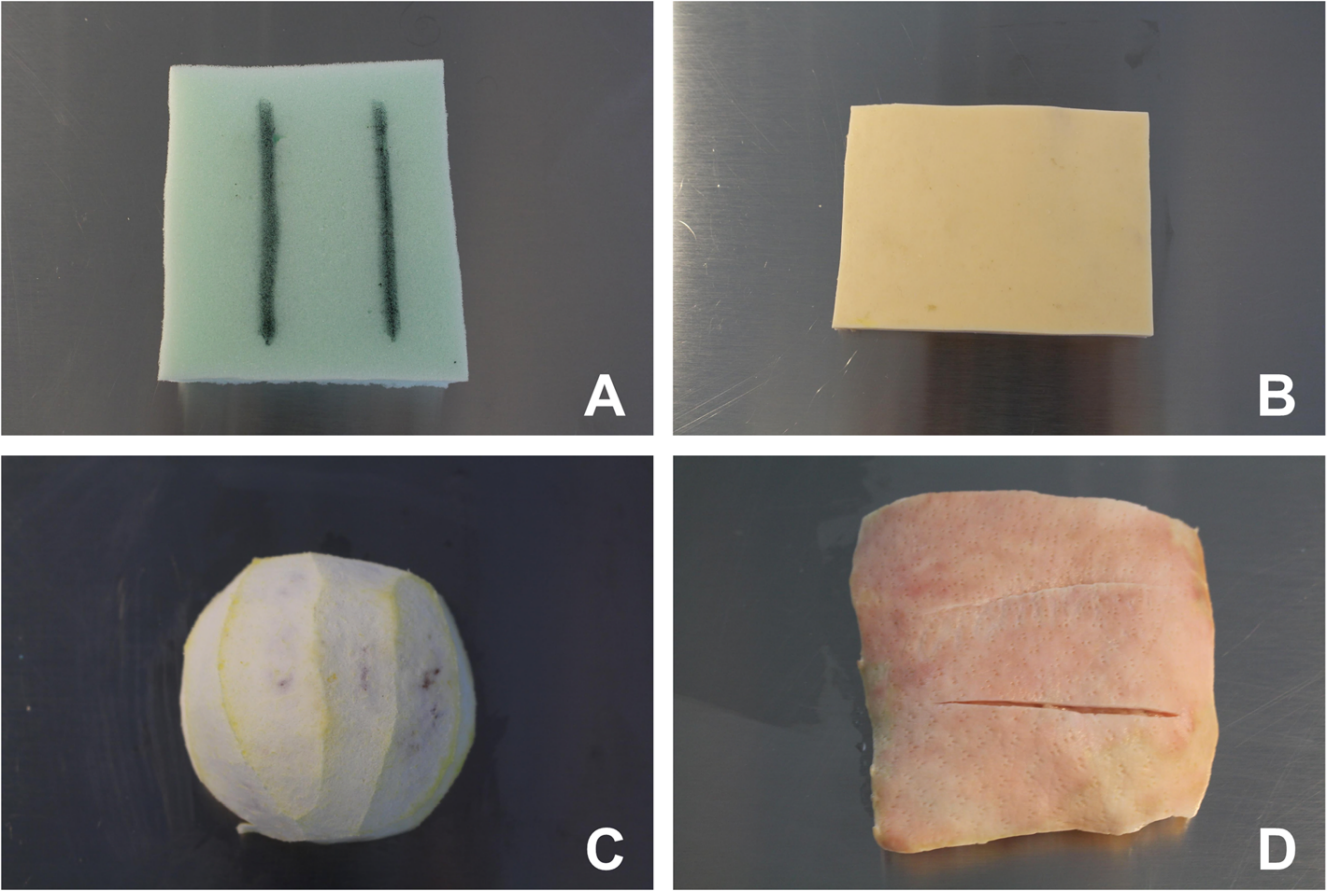
Suture models used from each category. **a** plant-based (peeled orange); **b** animal-based (a 6 × 6 in. segment of 1–1.5 in. thick pork fat skin); **c** synthethic-based (a 6 × 6 in. square of 1.5 in. thick dry sponge); **d** commercial-based (a 5 × 5 in. square of 0.7 in. thick multilayer silicon-rubber pad produced by the University’s Biomedical Engineering Department that simulates muscle, subcutaneous, and skin layers)

Four different categories of materials were chosen for the objective of this study: plant- (orange, grape, mango, banana, and eggplant), animal- (pork fat skin, chicken breast, pig foot, and euthanized laboratory rat [lethal dose of pentobarbital]), synthetic- (sponge, foam sheet, and Styrofoam), and commercial-based (artificial skin-like pads and silicon-rubber platform produced by the University) materials. The authors internally evaluated different models from each category and selected one based on their cost, availability, and qualities (Fig. 1).

### Results measurement

The study was explained to the participants, providing informed consent for signing. Those who accepted continued to a 1-h theory class taught by an expert educator with mastery in surgery. Basic suture information, suturing techniques (interrupted and continuous stitching), and knot-tying were taught and reviewed. Subsequently, the students were randomized and divided into groups, assigned to manipulate and practice suturing on each of the four models in a random order for 15-min intervals with feedback from instructors (phase 1).

In the experiment (phase 2), participants were randomly assigned to one of four groups. Each group had several samples of the same SM to equally balance each SM with the same number of students on their first to the fourth model. They had to perform three simple interrupted stitches and three-stitch continuous suture with a nylon 2–0 USP premium reverse cutting needle suture. The time needed to complete the task was registered and the quality of the knots was evaluated by the instructors (inter-rater reliability kappa coefficient of 0.86). The participants evaluated the SM after finishing the task (Supplement file 1). Students would then move to the next group to repeat with the following SM until completing all four.

Quality of stitches evaluation was designed between surgical experts with a Delphi method (Supplement file 2). Using a 3-point scale (1: deficient, 2: adequate, 3: ideal), the symmetry, firmness, and tension of the stitches were evaluated in each SM through the following stitching parameters: A) insertion and exit points were on the same plane; B) distance between the incision and insertion and exit points were similar; C) the length of the suture was similar to the distance between each one; D) knot firmness did not loosen with light/moderate manipulation; E) adequate suture tension.

The SM were evaluated by the participants using a 7-point Likert scale (in which 1 meant “Totally agree” and 7 meant “Totally disagree”) designed by the authors using the Delphi method. The questionnaire evaluated the material: A) was easy to handle and manipulate; B) had a consistency favorable for suturing practice; C) had adequate resistance and durability for practice; D) was suitable to make simple and continuous stitches; E) was adequate for learning BSS; F) was comfortable, clean, and hygienic to handle; G) inspires confidence to suture living human skin. Furthermore, two additional questions were placed for participants who had experience suturing living human skin in clinical scenarios: H) the SM simulates real human skin; I) the SM simulates the degree of difficulty of suturing human skin.

### Ethical considerations

The study was previously reviewed and approved by the ethics and research committees of the Hospital Universitario “Dr.Jose Eleuterio Gonzalez” of the Universidad Autónoma de Nuevo León’s with the registration number AH19–00007, certifying that it adheres to the guidelines of the General Health Law on Health Research in Human Beings of our country, as well as international guidelines and the Helsinki declaration. Human participation was voluntary. The experiment was explained to all participants allowing for questions or comments. Signed written informed consent was obtained from all participants, which could be withdrawn at any moment by the individual. Data and identity were confidential and only used for the purposes of this study. Personal protective equipment and materials (laboratory coat, medical gloves, facemask, hair net, sutures, and suturing instruments) were provided for all participants, by the laboratory. Plant-based materials used were fruits and vegetables obtained from a certified local market and taken to the laboratory to be washed and sanitized. Once oranges were internally chosen, these were obtained through the same process and pealed to leave halves for the experiment. After their use, these were placed in yellow bags and disposed of following the medical school’s laboratory guidelines. Animal-based materials were fresh pieces obtained from a certified local meat market, and taken to the laboratory to be sanitized and used for internal evaluation. One euthanized laboratory rat was obtained from the University to be evaluated as a model. It was obtained and regulated in accordance and approval of the Internal Committee for the Care and Use of Laboratory Animals of the Hospital Universitario “Dr.Jose Eleuterio Gonzalez” of the Universidad Autónoma de Nuevo León’s, which adheres to relevant national (NOM-062-ZOO-1999) and international law and guidelines (ARRIVE guidelines). All animal-based models once used, were placed in red bags and frozen at − 7 °C, until disposed of following the medical school’s laboratory guidelines. Pork-fat skin was chosen as the model, obtained through the same process, and managed likewise after the experiment.

### Analysis of data

The descriptive analysis of the qualitative variables was carried out using frequencies and percentages; quantitative variables are reported using median and interquartile range (IQR) after evaluating the distribution of the data using the Kolmogorov-Smirnov test. Comparisons between participants’ scores in the evaluations of their respective techniques were performed using the Kruskal-Wallis test and post hoc analysis with Bonferroni correction. Nominal variables were evaluated with a chi-square test.

A *p*-value < 0.05 was considered statistically significant. The Statistical Package for the Social Sciences (SPSS Statistics) version 24 (IBM, Armonk, NY, USA) for Windows 10 was used for statistical analysis, and the GraphPad Prism version 6 (GraphPad Software, San Diego, CA, USA) for graphic making.

## Results

A total of 82 MD students were recruited, of which 66 (80.5%) had no previous suturing experience on patients (median semester 4, IQR 2–6) while 16 (19.5%) had previous suturing experience (median semester 8, IQR 4–9.75). Ten students were from the first semester, who had not been exposed to the suturing workshop, part of the laboratory of Human Anatomy (a second-semester course). A significant difference (*p* < 0.001) was obtained for the semester grade between groups.

### Cost

The cost for a developing BSS could vary depending on different factors such as the SM, materials needed, extras with the use of biological tissues, and the environment of the workshops. The same materials and environment were used in this study, therefore cost analysis pertains only to the SM (Table 1). The low-fidelity models (oranges and dry sponge) were cheaper in comparison to the high-fidelity models (pork fat skin and silicon rubber pad).

**Table 1:** Suturing model cost and frequency of use

Material	Cost (USD)^a^	Number of uses	Pros	Cons
Orange	0.20	1	Accessible Low cost	Low endurance Juice - messy One use Low resistance Heterogeneuos consistency
Pork fat skin	1.40	7–12	Similarity to human tissue Consistency with skin True layers Better strength use perception	Refrigeration needed Biological material Time limit Smell Greasy Restricted access
Dry sponge	0.56	5–10	Accessible Low cost No time limit Easy storage Clean	Light weight, needs to be held down Poor depth perception Medium resistance
Silicon-rubber pad	5.60	10–15	Accessible No time limit Easy storage Generates self-percieved tension	Commercial brands at higher cost

*USD* united states dollars; ^a^ values calculated from Mexican pesos estimated at cost per participant

### Task completion time

**Fig. 2:**
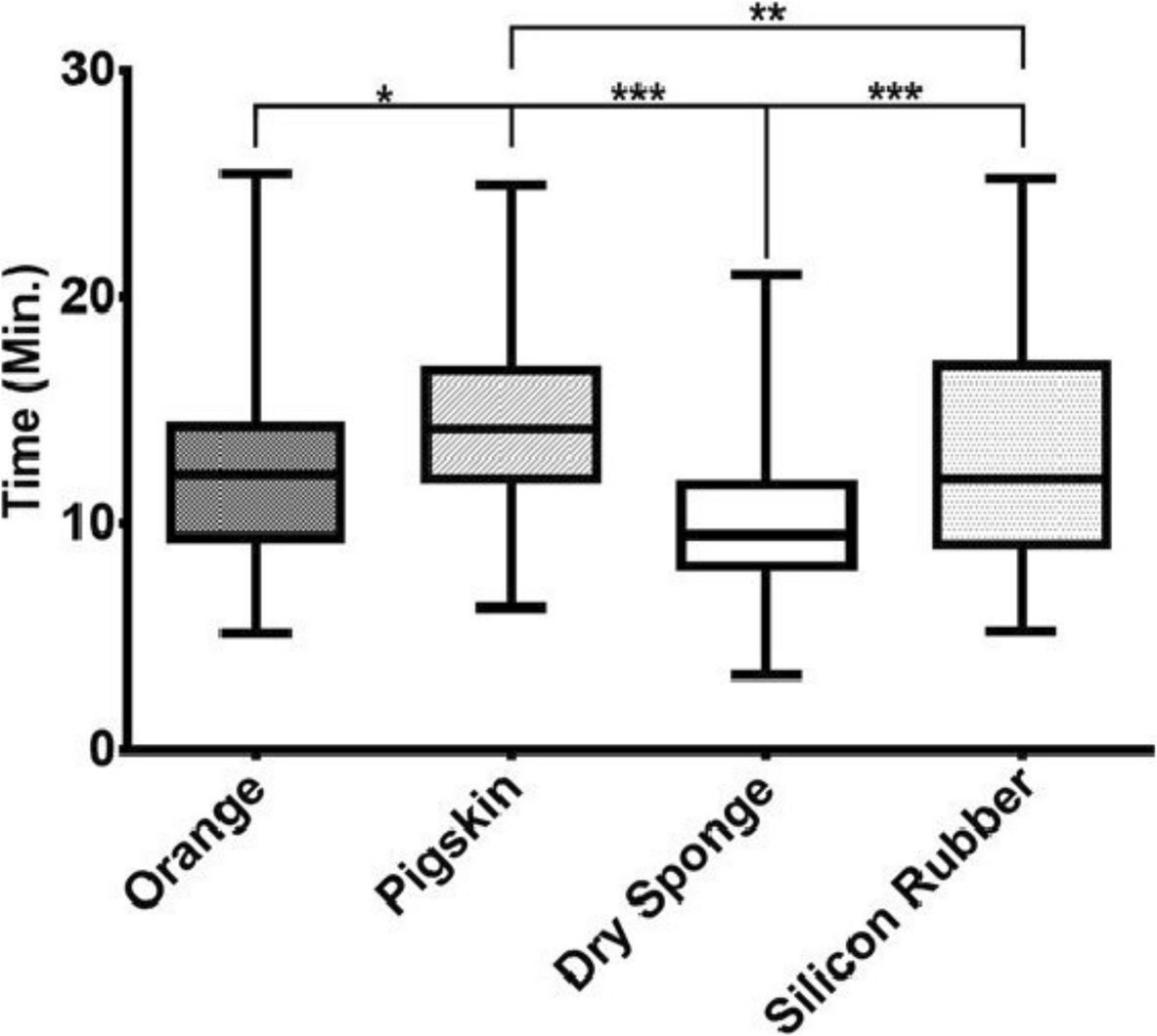
Box and whiskers graph of task completion time in each suturing model. Statistical analysis performed with Kruskal-Wallis test for independent samples with post-hoc analysis. * *p* < 0.05; ** *p* < 0.01; ***p < 0.001

The SM showed significant differences concerning task completion time. The dry sponge had the shortest median time (9.55 min), while the pork fat skin had the longest (14.39 min) (Table 2). These results showed significant differences (*p* < 0.001) when examined with the Kruskal-Wallis test for independent samples with posthoc analysis between SM (Table 3, Fig. 2). In Fig. 2 we can see the corresponding graph with these results.

**Table 2:** Task Completion Time in novice and experienced Medical Students

Suturing Model	Median (IQR)	Previous Suturing Experience Median (Interquartile range)		***P*** Value
		Yes	No	
**Orange**	12.2 (9.41–14.3)	10.43 (9.43–12.8)	12.28 (9.42–14.4)	0.358
**Pork Fat Skin**	14.39 (12.0–18.09)	13 (10.69–14.46)	15.1 (12.15–18.5)	< 0.05
**Dry Sponge**	9.55 (8.19–12.0)	8.7 (7.41–9.43)	10.06 (8.3–12.0)	< 0.05
**Silicon Rubber Pad**	12.02 (9.13–17.13)	9.94 (8.3–12.2)	12.15 (10.1–18.12)	< 0.05

Values expessed in minutes. Statistical analysis with Mann Whitney-u test

**Table 3:** Task Completion Time Group Comparison

	Pork fat skin	Dry sponge	Silicon-rubber pad
**Orange**	< 0.001	< 0.05	< 0.999
**Pork fat skin**		< 0.001	< 0.01
**Dry sponge**			< 0.001

Values expressed as p. Significance set a *p* < 0.05. Statistical analysis performed with Kruskal-Wallis test for independent samples with post-hoc analysis

The SM showed significant differences concerning task completion time regarding skill level. The dry sponge had the shortest median time in both the experienced (8.7 min) and novice students (10.06 min), while the pork fat skin had the longest (10.43 and 12.28 min, respectively). Completion time difference between participants with prior suturing experience and novice was evident in all models, except the orange (*p* = 0.358) (Table 2).

### Participants’ preference for suturing models

**Fig. 3:**
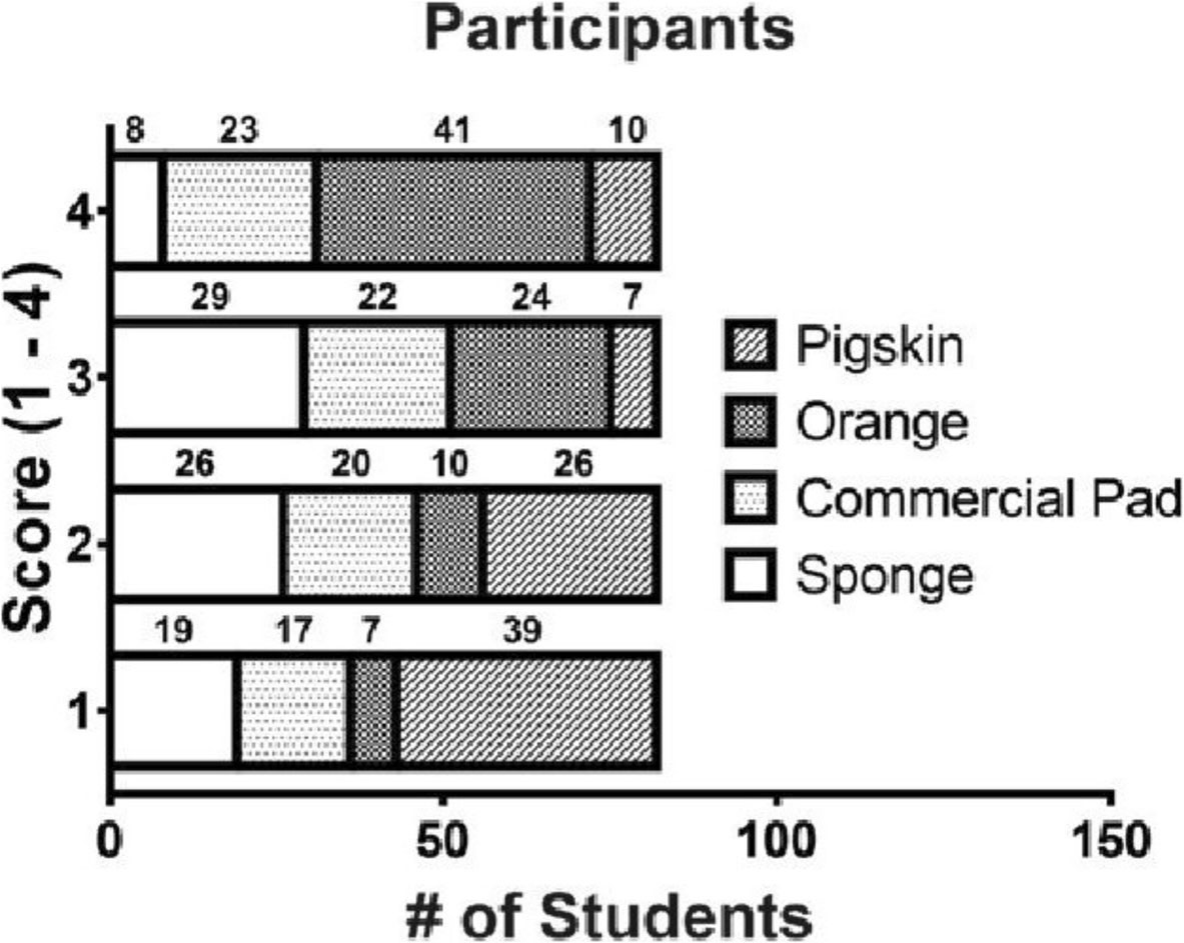
Suturing model preference. Participants were asked to order suturing models from their favorite (1) to least favorite (4) after completing all tasks. Stacked graph. Student preference for each suturing model. Number 1 was considered the best model and number 4 considered the worst. The exact number of students is reported above each segment of the stacked bar. Chi-Square test reported statistical significance *p* < 0.001

Participants were asked to evaluate each SM after completing the suturing task. After completing all models, participants were asked to list these in order according to their preference ([1] most favorite, [4] least favorite). Pork fat skin was considered the favorite SM (47.6% as the most favorite [1], and 31.7% as the second most favorite [2]) followed by the dry sponge (23.2% as the most favorite [1], and 31.7% as the second most favorite [2]). Silicon-rubber pad (20.07% [1] and 24.39% [2]), and orange (8.5% [1] and 12.19% [2]) followed in preference (Fig. 3). A chi-square showed statistical differences between the preference for suturing models (*p* < 0.001).

The 7-point Likert scale was used to compare satisfaction for each model (Table 4). Kruskal-Wallis test for independent samples showed significant differences in all the items for each model (*p <* 0.001). The SM with the highest mean scores were the dry sponge and the pork fat skin. The lowest was the peeled orange, which obtained the lowest results in all parameters.

**Table 4:** Likert confidence and satisfaction scale. Evaluation of the student’s perception of the different suturing models

The suturing model material:	Suturing Models (Median, IQR)				***P***-value
	Orange	Pork fat skin	Dry Sponge	Silicon-rubber pad	
Was easy to handle and manipulate	4 (2–5)	3 (2–4)	1 (1–2)	3 (1–4)	< 0.001
Had a consistency favorable for suturing practice	4 (3–6)	3 (2–4)	2 (1–3)	3 (2–5)	< 0.001
Had adequate resistance and durability for practice	5 (4–7)	1 (1–2)	3 (2–5)	4 (3–5)	< 0.001
Favorable to practice simple suture technique	3 (2–5)	2 (1–3)	2 (1–3)	3 (1–4)	< 0.001
Favorable to practice continuous suture technique	4 (2–5)	2 (1–3)	2 (1–3)	3 (1–5)	< 0.001
Was adequate for learning BSS	4 (2–5)	2 (1–3)	2 (2–3)	3 (1–4)	< 0.001
Was comfortable, clean, and hygienic to handle	4 (2–5)	3 (2–5)	1 (1–2)	2 (1–3)	< 0.001
Inspires confidence to suture living human skin	5 (3–7)	2 (1–3)	3 (2–5)	3 (2–5)	< 0.001
**Likert scale mean score**	4 (2.88–5.25)	2 (1.75–3.13)	2 (1.63–2.88)	3 (2–4.25)	< 0.001

7-point Likert scale in which 1 meant “Totally agree” and 7 meant “Totally disagree”. Statistical analysis performed using Kruskal-Wallis test with significance set a *p <* 0.05. IQR: interquartile range

A dry sponge was favored by its ease in manipulation, and consistency for suturing, over other SM, while also being a material that was clean, hygienic, and adequate for BSS learning. However, it was surpassed by the pork fat skin in resistance/durability and as a model that increases confidence for real patient suturing. Both were considered highly favorable for practicing simple and continuous stitching. IQR were ranged primarily between neutral and favorable results, indicating neither had a negative impression on the participants (Table 4).

Post hoc analysis determined there were no significant differences in the participants’ perception regarding consistency between the dry sponge and pork fat skin (*p* < 0.999), nor between the pork fat skin and the silicon-rubber gel pad (*p <* 0.999).

The most suitable model for acquiring BSS was the dry sponge, followed by pork fat skin, the silicon-rubber pad, and the orange. The Kruskal-Wallis test with Bonferroni correction found significant differences (*p* < 0.001) between each SM, except between dry sponge vs. pork fat skin, and orange vs. silicon-rubber pad (*p* < 0.999).

The cleanliness and hygiene were highest in the SM made from synthetic materials (dry sponge and silicon-rubber pad), and lower in the organic material models (orange and pork fat skin). The post hoc analysis showed no significant differences between the two synthetic models (*p <* 0.999), but there is significance between each of these models evaluated separately against the two organic models (*p <* 0.001).

Pork fat skin was the highest SM to inspire confidence among participants, followed by the sponge and commercial pad. These three models obtain positive scores for BSS acquisition, while orange is the only model with a negative score.

Two additional questions were included for participants with previous experience suturing patients. The first one was the similarity between the model and living human skin, for which pork fat skin was qualified as the most similar model (3 [2–4]) and the orange was the least (6 [5–7]). The second additional question was about the difficulty of suturing a determined model compared with human skin. Pork fat skin was the most similar in difficulty (3 [2–4]) with a slight difference with the silicon rubber pad (3 [3, 4]). The dry sponge (6 [5–7]) and the orange (6 [4–7]) had similar results in the second question, demonstrating a clear separation in the perception of the SM.

A statistically significant difference in both questions (*p <* 0.05) was identified with the Kruskal-Wallis test. The Bonferroni post hoc for the first question showed a significant difference (*p <* 0.05) between each model, except between the pork fat skin vs. silicon rubber pad, which may be due to the similarities of the two models.

The post hoc analysis of the second question only showed a significant difference (*p <* 0.05) between pork fat skin vs. dry sponge, and silicon rubber pad vs. dry sponge. The rest of the comparison between models did not have a statistical difference. This may be due to the pork fat skin and the silicon rubber pad obtaining similar high scores, likewise, the orange and dry sponge with similar low scores.

### Suturing quality evaluation in relation to suturing models

The scores obtained for each SM are higher in interrupted sutures than the continuous, technique (Table 5). This indicates the continuous stitches have an increased difficulty. Suture quality is influenced by SM characteristics. Statistically significant difference was similar between models in parameters B and D for both techniques, while only significant in the continuous technique for parameters A and C.

**Table 5:** Quality sutures scale for simple and continuous sutures. Kruskal-Wallis test reported statistical significance (**p < 0.05) (***p < 0.001)

Suturing Parameters			Suturing Models (Median, IQR)				***P***-value
			Orange	Pork fat skin	Dry Sponge	Silicon-rubber pad	
**Interrupted stitches**	**A**	Insertion and exit points were on the same plane	2 (2–3)	3 (2–3)	3 (2–3)	2 (1–3)	0.053
	**B**	Distance between the incision and insertion and exit points were similar	2 (1–2)	2 (2–3)	2 (2–3)	2 (1–2)	< 0.001***
	**C**	Length of the suture was similar to the distance between each one	2 (1–3)	2 (2–3)	2 (2–3)	3 (2–3)	0.219
	**D**	Knot firmness did not loosen with light/moderate manipulation	2 (1–3)	3 (2–3)	2 (1–3)	2 (2–3)	< 0.05**
	**E**	Adequate suture tension	2 (1–3)	3 (2–3)	2 (2–3)	3 (2–3)	0.113
	**F**	**Mean of quality scale**	**2.2 (1.6–2.4)**	**2.4 (2–2.8)**	**2.4 (1.8–2.8)**	**2.4 (1.8–2.6)**	< 0.05**
**Continuous stitches**	**A**	Insertion and exit points were on the same plane	2 (1–3)	2 (2–3)	3 (2–3)	2 (1–3)	< 0.05**
	**B**	Distance between the incision and insertion and exit points were similar	2 (1–2)	2 (2–2)	2 (2–3)	2 (1–2)	< 0.001***
	**C**	Length of the suture was similar to the distance between each one	2 (1–3)	2 (2–3)	2 (2–3)	2 (2–3)	< 0.05**
	**D**	Knot firmness did not loosen with light/moderate manipulation	2 (1–3)	2 (1–3)	2 (2–3)	2 (2–3)	< 0.05**
	**E**	Adequate suture tension	2 (1–3)	2 (1–3)	2 (1–3)	2 (2–3)	0.106
	**F**	**Mean of quality scale**	**1.8 (1.4–2.4)**	**2.2 (1.8–2.6)**	**2.2 (1.8–2.6)**	**2 (1.8–2.4)**	< 0.05**

3-point scale (1: deficient, 2: adequate, 3: ideal); A) insertion and exit points were on the same plane; B) distance between the incision and insertion and exit points were similar; C) the length of the suture was similar to the distance between each one; D) knot firmness did not loosen with light/moderate manipulation; E) adequate suture tension

Post-hoc analysis for interrupted sutures showed statistically significant differences (*p* < 0.05) between models in parameter B except between the pork fat skin vs. dry sponge. In parameter D, there was only a significant difference only between orange vs. pork fat skin (*p <* 0.05). In the continuous suturing techniques, these differences (*p <* 0.05) were only identified between the orange vs dry sponge in parameter A, between the dry sponge and silicon rubber pad in parameter B, and between orange and pork fat skin in parameter D.

These results evidence quality and probable difficulty between interrupted and continuous suturing. The tension-evaluating parameter (E) had no difference between all the different SM, which could be explained due to the lack of developed self-perception and motor skills in the students, explained as a time-dependent parameter, corrected by more time-effective practice.

### Experience

The comments analysis for each material highlighted some inherent factors in each model that influenced the acquisition of BSS. Participants mentioned characteristics in relation to the handling of the different materials at the time they made sutures, such as fragility, consistency, and hardness. These were free-handed comments made by students about the SM.

#### Orange

Was considered a novel material and accessible, but at the time of the practice they commented it was very fragile to handle, it tears easily and could become sticky because of the spilled juice. It was very easy to make the knots, although it was contrasted with parameter D. Students who had previously sutured on living human skin reported this model as very different from human skin, however, they did not make negative statements.

#### Dry sponge

was reported as a very comfortable material, easy to manipulate and suture, highlighting in the entrances and exits of the suture, although it is not similar to human skin. Students report that it is a good model for learning the initial suture techniques, however, they mention the difficulty in exerting proper tension on the knots.

#### Silicon rubber pad

Observations had bimodal distributions. The students mentioned it as an easy handling SM and simple to make the stitches. On the other hand, they refer the material was hard, making needle perforation and proper knot tension difficult. The resistance and consistency of this model created friction with the suture thread.

#### Pork fat skin

was reported as a very good model for acquiring BSS, and was frequently referred to as a favorite. It resembles human skin, and those with experience mentioned the possibility of practicing more advanced suture techniques. The most prevalent negative characteristic was the hardness/thickness of the skin, and the fat of the tissue made the instruments greasy, making it slower and more complicated to perform the stitches. This can be reflected in the time of task completion.

## Discussion

A wide variety of suturing training models have been described [6, 7, 9, 11–13]. Our study evaluates the user perception of BSS acquisition and suturing technique quality between different types of models among M.D. students.

The durability and resistance were evaluated in each model. The pork fat skin and the dry sponge were the best rated, coinciding with better scores obtained on the quality scale. There was a notable preference by the participants for the pork fat skin model, which is the model with the highest fidelity (Fig. 3). It also had high suturing quality scores. This may be due to its intrinsic characteristics such as tissue consistency, hardness, and similarity to human tissue, which helped students to obtain better outcomes. However, a Kruskal-Wallis test resulted in differences in the distance between insertion and exit points and knot firmness, suggesting not all parameters are influenced by the consistency and durability of the SM. Other parameters such as the tension on the stitches, quality of the knot, and the distance between each stitch are influenced by the strength needed to manipulate the material and the perception of the length and depth of the wound.

The dry sponge, although scored similarly to the pork fat skin, it was frequently selected as a second or third preferred SM (Fig. 3), and scored lower in confidence/motivation (Table 4). The analysis demonstrates a good/high opinion for a determined model does not necessarily correspond with a high fidelity model, but rather it is related to self-perception. This may be due to the SM characteristics, such as the tensile strength and the resistance and durability of the tissue. These characteristics are cited among different authors as advantages in a model [14–16]. Although these factors are not studied individually, they are considered as a part of each model.

The peeled orange could not be adequately manipulated by the participants due to its characteristics, which in turn reflected in the dissatisfaction of the model. The organic material was fragile and easy to tear, resulting in low-quality stitches in the evaluation (experimental observation). This indicates the intrinsic characteristics of the model material such as consistency and resistance are important in the selection of a SM for training.

The resistance was an important SM factor for quality stitches and knots. There are scarce studies that take this variable into account [17], focusing primarily on strength or resistance [15, 16]. Increase exposure to practice sessions could improve the results for these parameters [18].

In numerous studies, the role of animal and synthetic suture models in the acquisition of BSS has been evaluated, among them it has been seen that there are no differences between the performance of students with synthetic or animal models [19, 20]. However, other studies have shown greater confidence in the skills acquired, and a better quality of suture stitches with animal models [21]. As that may be, the reduced access to animal materials must be taken into consideration. Vegetable models have been criticized due to their thin and friable structure, which increases the difficulty in the acquisition of BSS [22]. Table 1 discusses the advantages and disadvantages that each of the models used may have.

### Fidelity of suture models

Our results evidence model fidelity plays an important role in the students learning and confidence acquisition, suggesting an essential role in BSS training. However, published studies have contradictory data, [12–14, 23] some reporting no differences, [14] or even a negative correlation [24]. A greater amount of studies in this area are needed to establish conclusive data [25].

### The importance of the model in the acquisition of BSS

Selecting the best SM to develop BSS in students, several key aspects must be considered. The cost, accessibility, maintenance, storage, durability, duration period, the need for special equipment/installations, skill objectives, and other characteristics such as those of the suture (size, type of needle, configuration, elasticity, memory), type of suturing technique taught, and experience of the user [26, 27]. Advanced technique training such as biopsies [5, 6], aesthetic skin wound closure [7, 9], basic Z-plasty [12], Mohs technique [28] or grafts and flaps [29], require organic models in which tissue layers are well defined, simulate real characteristics, and the tissue can move freely, allowing the trainee to perform these techniques almost identically to that of a patient [30].

The users’ perception of a model influences in defining it as a competent model or not for BSS. This also correlates to the participant’s confidence to perform surgical skills in patients in a safe approach [21]. A positive self-perception of their performance can strengthen the skills learned [31]. Although this was evidenced between models in the student perception analysis, this was limited by the time available during the experimental practice. A prospective design between low- and high-fidelity models could aid in determining SM impact on their skill confidence.

Teaching sessions where students are trained to suture patients have been shown to generate more confidence in them. These effects have been seen both in the short term, with training immediately before starting the internship, and in the long term in sessions taught in the first years of medical school [3]. This is also the case in other necessary skills in the surgical branch that do not directly involve suturing a patient [32]. Manning et al. carried out a study where first-year medical students were taught to suture in the anatomy laboratory. Subsequently, the students’ confidence was evaluated during their clerkship demonstrating increased confidence as well as a higher number of patients sutured during their clinical practice [31]. Supervised suture sessions have a long-term impact on the student, however, human cadavers availability may differ between schools and countries, as well as the conditions of the tissues, depending on the preservation techniques [33–38]. A high volume of students, as is the case in many schools in Latin America, also raises variables to consider when teaching BSS [39–41]. This is why is necessary for other alternatives that allow developing these skills.

### Limitations of the study

All medical students are exposed to suturing courses during their second semester of medical school, however additional exposure to suturing courses was not evaluated in our participants. Only ten students had not been exposed to the standard suturing workshop. The confidence and skill may vary due to the difference in semesters of medical school, but was most evident by the experience of previously suturing patients with shorter task completion time. A premium reverse cutting needle was used in all SM which could influence de durability of the materials. A taper point needle could have produced different results in the less resistant materials. The practice time was also finite, limiting the skill development practice, before the experiment. The transverse design does not allow monitoring skill and confidence retention beyond the suturing session. Costs are expressed in United States Dollars (USD) converted from Mexican Pesos (MXP). The costs of materials and availability may be different in other countries, especially with animal-based materials. The multilayered silicon-rubber pad was made by the biomedical engineering department at the university, reducing the cost to approximately $5.60 USD per pad, cheaper than commercial pads which can range between $15 and $55 USD per pad. Future studies should also focus on developing improved and effective synthetic materials that may be comparable and affordable for users, to benefit those with limited access to animal-based materials. The evaluation of SM variables may differ between commercial pads vs. the University made pad.

The strengths of the study include a large number of participants suturing in each of the four SM in a randomized order.

## Conclusions

Medical students need an adequate level of BSS to safely care for patients. These skills can be adequately learned in low-cost models that allow the student to safely experience human skin with reasonable resemblance. Since the courses to learn and practice BSS can be carried out by different groups in relatively short periods, animal models such as pork fat skin or animal pieces can be used, which can be kept for these periods without generating hygienic problems and also allow the correct performance of techniques. We can conclude that globally, orange is a model with a greater number of deficiencies than the rest of the SM evaluated. Both, dry sponge and pork fat skin have similar efficiency and BSS development, with a higher preference by students for the pork fat skin.

## Supplementary Information

**Additional file 1.**

**Additional file 2.**

## Data Availability

All data generated or analysed during this study are included in this published article and its supplementary information files. Any other data can be made available upon reasonable request.

## Abbreviations

BSS: Basic surgical skills
SM: Suturing models
IQR: Interquartile range
MD: Medical degree
USD: United States Dollars
MXP: Mexican Pesos
