# Supplementary material for: Comparison of suturing models: the effect on perception of basic surgical skills
Source: BMC Med Educ. 2021 May 1;21:250. doi: 10.1186/s12909-021-02692-x (PMC8088011; doi:10.1186/s12909-021-02692-x)
Supplement: Supplementary file 1 — Additional file 1. [file 12909_2021_2692_MOESM1_ESM.docx]

**Comparison of suturing models: the effect on perception of basic surgical skills**

Alejandro Rafael Gonzalez-Navarro^1β^, Alejandro Quiroga-Garza^1,2β^, Adriana Sharai Acosta-Luna^1^, Yolanda Salinas-Alvarez^1^, Javier Humberto Martinez-Garza^1^, Oscar De la Garza-Castro^1^, Jorge Gutierrez-de la O^1^, David de la Fuente-Villarreal^1^, Rodrigo Enrique Elizondo-Omaña^1^*, Santos Guzman-Lopez^1^*

Institutional Affiliations:

^1^ Universidad Autonoma de Nuevo Leon, School of Medicine, Human Anatomy Department, Monterrey, Nuevo Leon, Mexico.

^2^ Instituto Mexicano del Seguro Social, Delegación de Nuevo Leon, General Surgery, Monterrey, Nuevo Leon, Mexico.

^β^ both authors participated equally in the study, and are both in the position of first author, by alphabetical order.

***Corresponding author**

Santos Guzman-Lopez and Rodrigo Enrique Elizondo-Omaña

Universidad Autónoma de Nuevo León, Facultad de Medicina, Departamento de Anatomia Humana. Francisco I. Madero and Jose E. Gonzalez sin número, Colonia Mitras Centro Monterrey, Nuevo León, México, 64460

Telephone: +52 81 83- 29-41-71

e-mail: [pattyg19@gmail.com](mailto:pattyg19@gmail.com) and [rod_omana@yahoo.com](mailto:rod_omana@yahoo.com)

**Confidence and Satisfaction Scale**

**Student initials: ________________ Sex: ________________** **Semester:_______**

1. Have you previously received suturing workshops or courses? (excluding the one given in the anatomy course)

**Yes No**

1. Have you sutured human skin in a clinical scenario?

**Yes No**

**Orange** Suturing Model

Number in Randomization:_______ Time: _______

The suturing model material:

|  | Totally Agree | Agree | Partially Agree | Neutral | Partially Disagree | Disagree | Totally Disagree |
| --- | --- | --- | --- | --- | --- | --- | --- |
| Was easy to handle and manipulate | 7 | 6 | 5 | 4 | 3 | 2 | 1 |
| Had a consistency favorable for suturing practice | 7 | 6 | 5 | 4 | 3 | 2 | 1 |
| Had adequate resistance and durability for practice | 7 | 6 | 5 | 4 | 3 | 2 | 1 |
| Favorable to practice simple suture technique | 7 | 6 | 5 | 4 | 3 | 2 | 1 |
| Favorable to practice continuous suture technique | 7 | 6 | 5 | 4 | 3 | 2 | 1 |
| Was adequate for learning BSS | 7 | 6 | 5 | 4 | 3 | 2 | 1 |
| Was comfortable, clean, and hygienic to handle | 7 | 6 | 5 | 4 | 3 | 2 | 1 |
| Inspires confidence to suture living human skin | 7 | 6 | 5 | 4 | 3 | 2 | 1 |
| Answer the following questions **ONLY** if you answered “**YES**” to **Question #2** | | | | | | | |
| Simulates real human skin | 7 | 6 | 5 | 4 | 3 | 2 | 1 |
| Simulates the degree of difficulty of suturing human skin | 7 | 6 | 5 | 4 | 3 | 2 | 1 |

Additional Comments:

________________________________________________________________________________________________________________________________________________________

**Pork Fat Skin** Suturing Model

Number in Randomization:_______ Time: _______

The suturing model material:

|  | Totally Agree | Agree | Partially Agree | Neutral | Partially Disagree | Disagree | Totally Disagree |
| --- | --- | --- | --- | --- | --- | --- | --- |
| Was easy to handle and manipulate | 7 | 6 | 5 | 4 | 3 | 2 | 1 |
| Had a consistency favorable for suturing practice | 7 | 6 | 5 | 4 | 3 | 2 | 1 |
| Had adequate resistance and durability for practice | 7 | 6 | 5 | 4 | 3 | 2 | 1 |
| Favorable to practice simple suture technique | 7 | 6 | 5 | 4 | 3 | 2 | 1 |
| Favorable to practice continuous suture technique | 7 | 6 | 5 | 4 | 3 | 2 | 1 |
| Was adequate for learning BSS | 7 | 6 | 5 | 4 | 3 | 2 | 1 |
| Was comfortable, clean, and hygienic to handle | 7 | 6 | 5 | 4 | 3 | 2 | 1 |
| Inspires confidence to suture living human skin | 7 | 6 | 5 | 4 | 3 | 2 | 1 |
| Answer the following questions **ONLY** if you answered “**YES**” to **Question #2** | | | | | | | |
| Simulates real human skin | 7 | 6 | 5 | 4 | 3 | 2 | 1 |
| Simulates the degree of difficulty of suturing human skin | 7 | 6 | 5 | 4 | 3 | 2 | 1 |

Additional Comments:

________________________________________________________________________________________________________________________________________________________

**Dry Sponge** Suturing Model

Number in Randomization:_______ Time: _______

The suturing model material:

|  | Totally Agree | Agree | Partially Agree | Neutral | Partially Disagree | Disagree | Totally Disagree |
| --- | --- | --- | --- | --- | --- | --- | --- |
| Was easy to handle and manipulate | 7 | 6 | 5 | 4 | 3 | 2 | 1 |
| Had a consistency favorable for suturing practice | 7 | 6 | 5 | 4 | 3 | 2 | 1 |
| Had adequate resistance and durability for practice | 7 | 6 | 5 | 4 | 3 | 2 | 1 |
| Favorable to practice simple suture technique | 7 | 6 | 5 | 4 | 3 | 2 | 1 |
| Favorable to practice continuous suture technique | 7 | 6 | 5 | 4 | 3 | 2 | 1 |
| Was adequate for learning BSS | 7 | 6 | 5 | 4 | 3 | 2 | 1 |
| Was comfortable, clean, and hygienic to handle | 7 | 6 | 5 | 4 | 3 | 2 | 1 |
| Inspires confidence to suture living human skin | 7 | 6 | 5 | 4 | 3 | 2 | 1 |
| Answer the following questions **ONLY** if you answered “**YES**” to **Question #2** | | | | | | | |
| Simulates real human skin | 7 | 6 | 5 | 4 | 3 | 2 | 1 |
| Simulates the degree of difficulty of suturing human skin | 7 | 6 | 5 | 4 | 3 | 2 | 1 |

Additional Comments:

________________________________________________________________________________________________________________________________________________________

**Silicon-rubber pad** Suturing Model

Number in Randomization:_______ Time: _______

The suturing model material:

|  | Totally Agree | Agree | Partially Agree | Neutral | Partially Disagree | Disagree | Totally Disagree |
| --- | --- | --- | --- | --- | --- | --- | --- |
| Was easy to handle and manipulate | 7 | 6 | 5 | 4 | 3 | 2 | 1 |
| Had a consistency favorable for suturing practice | 7 | 6 | 5 | 4 | 3 | 2 | 1 |
| Had adequate resistance and durability for practice | 7 | 6 | 5 | 4 | 3 | 2 | 1 |
| Favorable to practice simple suture technique | 7 | 6 | 5 | 4 | 3 | 2 | 1 |
| Favorable to practice continuous suture technique | 7 | 6 | 5 | 4 | 3 | 2 | 1 |
| Was adequate for learning BSS | 7 | 6 | 5 | 4 | 3 | 2 | 1 |
| Was comfortable, clean, and hygienic to handle | 7 | 6 | 5 | 4 | 3 | 2 | 1 |
| Inspires confidence to suture living human skin | 7 | 6 | 5 | 4 | 3 | 2 | 1 |
| Answer the following questions **ONLY** if you answered “**YES**” to **Question #2** | | | | | | | |
| Simulates real human skin | 7 | 6 | 5 | 4 | 3 | 2 | 1 |
| Simulates the degree of difficulty of suturing human skin | 7 | 6 | 5 | 4 | 3 | 2 | 1 |

Additional Comments:

________________________________________________________________________________________________________________________________________________________

1. Overall, in which order of preference would you list the suturing models (1 Most Favorite, 4 Least Favorite)

Orange _____ Pork fat skin _____ Dry Sponge _____ Silicon-rubber pad _____

**Course satisfaction: (at the end of practice)**

|  | Totally Agree | Agree | Partially Agree | Neutral | Partially Disagree | Disagree | Totally Disagree |
| --- | --- | --- | --- | --- | --- | --- | --- |
| I was satisfied with the content of the theory part of the course regarding the teaching of basic sutures | 7 | 6 | 5 | 4 | 3 | 2 | 1 |
| The suturing course generated greater personal confidence for the development of suturing skills | 7 | 6 | 5 | 4 | 3 | 2 | 1 |
| Physicians must possess basic suturing skill | 7 | 6 | 5 | 4 | 3 | 2 | 1 |
| I feel capable of suturing human skin | 7 | 6 | 5 | 4 | 3 | 2 | 1 |
| Suturing courses increase my interest in surgical specialties | 7 | 6 | 5 | 4 | 3 | 2 | 1 |
