# Supplementary material for: Comparison of suturing models: the effect on perception of basic surgical skills
Source: BMC Med Educ. 2021 May 1;21:250. doi: 10.1186/s12909-021-02692-x (PMC8088011; doi:10.1186/s12909-021-02692-x)
Supplement: Supplementary file 2 — Additional file 2. [file 12909_2021_2692_MOESM2_ESM.docx]

**Comparison of suturing models: the effect on perception of basic surgical skills**

Alejandro Rafael Gonzalez-Navarro^1β^, Alejandro Quiroga-Garza^1,2β^, Adriana Sharai Acosta-Luna^1^, Yolanda Salinas-Alvarez^1^, Javier Humberto Martinez-Garza^1^, Oscar De la Garza-Castro^1^, Jorge Gutierrez-de la O^1^, David de la Fuente-Villarreal^1^, Rodrigo Enrique Elizondo-Omaña^1^*, Santos Guzman-Lopez^1^*

Institutional Affiliations:

^1^ Universidad Autonoma de Nuevo Leon, School of Medicine, Human Anatomy Department, Monterrey, Nuevo Leon, Mexico.

^2^ Instituto Mexicano del Seguro Social, Delegación de Nuevo Leon, General Surgery, Monterrey, Nuevo Leon, Mexico.

^β^ both authors participated equally in the study, and are both in the position of first author, by alphabetical order.

***Corresponding author**

Santos Guzman-Lopez and Rodrigo Enrique Elizondo-Omaña

Universidad Autónoma de Nuevo León, Facultad de Medicina, Departamento de Anatomia Humana. Francisco I. Madero and Jose E. Gonzalez sin número, Colonia Mitras Centro Monterrey, Nuevo León, México, 64460

Telephone: +52 81 83- 29-41-71

e-mail: [pattyg19@gmail.com](mailto:pattyg19@gmail.com) and [rod_omana@yahoo.com](mailto:rod_omana@yahoo.com)

**Quality of Stitches**

**Student initials: ________________ Sex: ________________** **Semester:_______**

**Instructions:** Mark in each number the score corresponding to each of the characteristics indicated with respect to the two types of knot. The number 1 represents the lowest score that can be obtained and the number 3 the highest.

Scale: 1: deficient, 2: adequate, 3: ideal

| # |  | Interrupted stitches | | |  | Continuous stitches | | |
| --- | --- | --- | --- | --- | --- | --- | --- | --- |
| A | Insertion and exit points were on the same horizontal plane | 1 | 2 | 3 |  | 1 | 2 | 3 |
| B | Distance between the incision and insertion and exit points were similar | 1 | 2 | 3 |  | 1 | 2 | 3 |
| C | Length of the suture was similar to the distance between each one | 1 | 2 | 3 |  | 1 | 2 | 3 |
| D | Knot firmness did not loosen with light/moderate manipulation. | 1 | 2 | 3 |  | 1 | 2 | 3 |
| E | Stitches had adequate suture tension | 1 | 2 | 3 |  | 1 | 2 | 3 |
|  | Total |  | | |  |  | | |

Representation of parameters evaluated in the first three questions of the survey:

x

B

x

A

2x

C
